# Outcomes and management approaches of resuscitative endovascular balloon occlusion of the aorta based on the income of countries

**DOI:** 10.1186/s13017-020-00337-w

**Published:** 2020-10-12

**Authors:** Ramiro Manzano-Nunez, David McGreevy, Claudia P. Orlas, Alberto F. García, Tal M. Hörer, Joseph DuBose, Carlos A. Ordoñez, on behalf of the AAST-AORTA Investigators and the ABOTrauma Registry Group, Jonny Morrison, Thomas M. Scalea, Laura J. Moore, Jeanette M. Podbielski, John B. Holcomb, Kenji Inaba, Alice Piccinini, David S. Kauvar, Valorie L. Baggenstoss, Catherine Rauschendorfer, Jeremey Cannon, Mark Seamon, Ryan Dumas, Mike Vella, Jessica Guzman, Chance Spalding, Timothy W. Wolff, Chuck Fox, Ernest Moore, David Turay, Cassra N. Arbabi, Xian Luo-Owen, David Skarupa, Jennifer A. Mull, Joannis Baez Gonzalez, Joseph Ibrahim, Karen Safcsak, Stephanie Gordy, Michael Long, Andrew W. Kirkpatrick, Chad G. Ball, Zhengwen Xiao, Elizabeth Dauer, Jennifer Knight, Nicole Cornell, Forrest Dell Moore, Matthew Bloom, Nam T. Tran, Eileen Bulger, Jeannette G. Ward, John K. Bini, John Matsuura, Joshua Pringle, Karen Herzing, Kailey Nolan, Nathaniel Poulin, William Teeter, Rachel Nygaard, Chad Richardson, Joseph Skaja, Derek Lombard, Reagan Bollig, Brian Daley, Niki Rasnake, Marko Bukur, Elizabeth Warnack, Joseph Farhat, Robert M. Madayag, Pamela Bourg, Anna Ramstedt, Mitra Sadeghi, Kristofer F. Nilsson, Thomas Larzon, Artai Pirouzram, Asko Toivola, Mariusz Maszkowski, Adam Bersztel, Per Skoog, Koji Idoguchi, Yuri Kon, Tokiya Ishida, Yosuke Matsumura, Junichi Matsumoto, Viktor Reva, Eva-Corina Caragounis, Mårten Falkenberg, Lauri Handolin, George Oosthuizen, Endre Szarka, Vassil Manchev, Tongporn Wannatoop, Sung W. Chang, Boris Kessel, Dan Hebron, Gad Shaked, Miklosh Bala, Federico Coccolini, Luca Ansaloni, Peter Hibert-Carius, Michelle Moe, Suzanne Maria Vrancken

**Affiliations:** 1 Clinical Research Center, Fundacion Valle del Lili, Cali, Colombia; 2 Universidad del Rosario, Escuela de Medicina y Ciencias de la Salud, Bogotá, Colombia; 3 Méderi Hospital Universitario Mayor, Carrera 24 No 63C - 69 Barrio Siete de Agosto, Bogotá, DC Colombia; 4 R. Adams Cowley Shock Trauma, Baltimore, MD USA; 5 Department of Surgery, Fundación Valle del Lili, Cali, Colombia; 6 Department of Cardiothoracic and Vascular Surgery, Faculty of Medicine and Health, Örebro University, Örebro, Sweden

**Keywords:** REBOA, Income of countries, Trauma

## Abstract

**Background:**

Resuscitative endovascular balloon occlusion of the aorta (REBOA) could provide a survival benefit to severely injured patients as it may improve their initial ability to survive the hemorrhagic shock. Although the evidence supporting the use of REBOA is not conclusive, its use has expanded worldwide. We aim to compare the management approaches and clinical outcomes of trauma patients treated with REBOA according to the countries’ income based on the World Bank Country and Lending Groups.

**Methods:**

We used data from the AORTA (USA) and the ABOTrauma (multinational) registries. Patients were stratified into two groups: (1) high-income countries (HICs) and (2) low-to-middle income countries (LMICs). Propensity score matching extracted 1:1 matched pairs of subjects who were from an LMIC or a HIC based on age, gender, the presence of pupillary response on admission, impeding hypotension (SBP ≤ 80), trauma mechanism, ISS, the necessity of CPR on arrival, the location of REBOA insertion (emergency room or operating room) and the amount of PRBCs transfused in the first 24 h. Logistic regression (LR) was used to examine the association of LMICs and mortality.

**Results:**

A total of 817 trauma patients from 14 countries were included. Blind percutaneous approach and surgical cutdown were the preferred means of femoral cannulation in HICs and LIMCs, respectively. Patients from LMICs had a significantly higher occurrence of MODS and respiratory failure. LR showed no differences in mortality for LMICs when compared to HICs; neither in the non-matched cohort (OR = 0.63; 95% CI: 0.36‑1.09; *p* = 0.1) nor in the matched cohort (OR = 1.45; 95% CI: 0.63‑3,33; *p* = 0.3).

**Conclusion:**

There is considerable variation in the management practices of REBOA and the outcomes associated with this intervention between HICs and LMICs. Although we found significant differences in multiorgan and respiratory failure rates, there were no differences in the risk-adjusted odds of mortality between the groups analyzed. Trauma surgeons practicing REBOA around the world should joint efforts to standardize the practice of this endovascular technology worldwide.

## Introduction

Resuscitative endovascular balloon occlusion of the aorta (REBOA) is becoming an appealing alternative in the resuscitation of exsanguinating patients with non-compressible torso hemorrhage (NCTH) [1]. Its use, as a tool within the broad concept of endovascular resuscitation and trauma management [2], has expanded worldwide [3–5], and data on trauma patients treated with REBOA from different countries in the world have been collected in different registries [6, 7]. Furthermore, the field of endovascular hemorrhage control has experienced significant growth in the past years, and literature has emerged that offers contradictory findings of its safety and effectiveness [1, 8–10].

Although REBOA is being used worldwide, there is evident variation in working conditions, workforce training, available health care systems, and economic resources across different countries. These factors may have a significant impact on the care, and surgical outcome of REBOA resuscitated patients. In order to address the variations between regions and countries in relation to their income, we aim to compare the management approaches and clinical outcomes of trauma patients resuscitated with REBOA according to the countries’ income based on the World Bank Country and Lending Groups.

## Methods

The data presented in this observational study represents a cohort of trauma patients resuscitated with REBOA worldwide.

### Data source

For this study, we used data from the AORTA and the ABOTrauma registries. These registries were launched to collect patient and outcome data from trauma patients treated with REBOA. Details of each registry (AORTA, ABOTrauma) have been outlined in previous publications [6, 7, 11, 12].

In brief, the AORTA registry is an initiative of the AAST Multicenter Trials Committee and collects data of adult trauma patients (age 18 or older) undergoing aortic occlusion (either by aortic cross-clamping or by REBOA) in the acute phases after injury and treated at level-I trauma centers within the continental USA [6]. The ABOTrauma registry is based on Sweden and collects data on patients who had REBOA for the management of traumatic hemorrhagic shock from 25 institutions at 13 countries from Europe, Asia, and South America [7].

The AAST Multicenter Trials Committee and the Regional Ethics Committee of Uppsala, Sweden, approved the protocols for the AORTA and ABOTrauma registry, respectively. Also, all contributing hospitals received ethical approval from their own ethics committees.

### Patient groups

Trauma patients undergoing REBOA were eligible for analysis. We planned to exclude patients undergoing REBOA for other indications different than trauma (i.e., non-traumatic hemorrhagic shock). Included patients were stratified depending on their classification in the World Bank Country and Lending groups [13]. In this classification, the World Bank divides the world’s economies into four groups: high, upper-middle, lower-middle, and low. However, due to the limited sample sub-group size, we decided to divide the patients into two groups: (1) high-income countries and (2) upper-middle, lower-middle, and low-income countries.

The data merged from both registries was from 14 countries: 9 high-income countries (the USA, Israel, Sweden, Finland, Japan, Italy, South Korea, Germany, and the Netherlands), four upper-middle-income countries (Russia, Thailand, Colombia, and Turkey), and one low-income country (North Korea).

### Variables and outcomes

Collected variables included demographic information, mechanism of injury, blood transfusion requirements, admission physiology, surgical interventions for bleeding control, REBOA access and deployment techniques, zone of the occlusion, organ failure, and mortality. Our primary endpoint was in-hospital mortality.

### Statistical analysis

After merging the data from both registries, patients were classified according to the World Bank Country and Lending group of their country of origin: low to middle (LMICs) vs. high-income countries (HICs). Data were first compared between LMICs and HICs groups. Results were presented using frequencies and percentages for dichotomous and categorical variables and medians and inter-quartile ranges for continuous variables. Statistical comparisons between the two groups were made using Wilcoxon rank-sum test for continuous variables and chi-square tests or Fisher’s exact tests in cell counts less than five for categorical variables.

Because REBOA resuscitated patients were not randomly assigned either to an LMIC or a HIC, a formal causal inference regarding the income of countries and the outcomes of interest is not possible. Therefore, to examine the contribution of the patient country of origin income, we generated a propensity score for each patient. For the propensity score, a logistic regression model was fit in order to predict each patient probability (propensity) of being from a LMIC or a HIC as a function of available covariates. These covariates were age, gender, the presence of pupillary response on admission, impending hypotension (SBP ≤ 80), trauma mechanism, ISS, the necessity of CPR on arrival, the location of REBOA insertion (emergency room or operating room), and the amount of PRBCs transfused in the first 24 h.

Propensity score matching extracted 1:1 matched pairs of subjects who were from an LMICs or a HIC. Within the cohort of matched individuals, logistic regression was used to examine the association of LMICs and mortality. Logistic regression was adjusted by tachycardia (HR ≥ 100), procedure (laparotomy or pelvic surgery), the value of systolic blood pressure at REBOA initiation, and the need for massive transfusion (≥ 10 PRBCs in the first 24 h). All analyses were performed in the Stata 14 Statistical Software.

## Results

A total of 817 trauma patients from 14 countries were treated with REBOA and included in the analysis.

Table 1 presents an overview of baseline characteristics and therapeutic strategies stratified by high and low-to-middle income countries, and the number of missing values for each variable. The median (IQR) of age was 42 (27‑58.5); 204/817 (25%) were female. Blunt trauma was the most common trauma mechanism (633/800; 79.1%), and patients were often victims of severe injuries (ISS, median [IQR]: 34 [25‑45]). Although there were no differences in severity scores between HICs and LMICs, patients from LMICs were less likely to receive pre-hospital and admission CPR. Also, a significantly higher proportion of them arrived with a systolic blood pressure of 80 mmHg or less, and with tachycardia (Table 1). Patients from HICs received significantly higher amounts of red blood cells and plasma and were more likely to undergo angioembolization for hemorrhage control (Table 1).

**Table 1:** Baseline characteristics, resuscitation strategies, and surgical interventions between LMICs and HICs

Variable	All (***n*** = 817)	LMIC (***n*** = 73)	HIC (***n*** = 744)	***p*** value	Missing
Age, median (IQR)	42 (27‑58.5)	37 (27‑49)	43.5 (27.5‑59.5)	0.08	13
Gender [female], *n* (%)	204 (25%)	19 (26%)	185 (24.9%)	0.8	0
**Trauma mechanism**				< 0.001	17
Blunt, *n* (%)	633/800 (79.1%)	42/73 (57.5%)	591/727 (81.2%)		
Penetrating, *n* (%)	167/800 (20.8%)	31/73 (42.7%)	136/727 (18.7%)		
ISS, median (IQR)	34 (25‑45)	32 (25‑50)	34 (25‑45)	0.6	96
Pre-Hosp CPR, *n* (%)	165/786 (21%)	1/71 (1.4%)	164/715 (22.9)	< 0.001	31
CPR on arrival, *n* (%)	137/789 (17.3)	3 /73 (4.1%)	134/18.7 (18.7%)	< 0.001	28
**Admission SBP**				< 0.001	30
Not measurable	149/787 (18.9%)	11/73 (15%)	138/714 (19.3%)		
< 50	58/787 (7.3%)	10/73 (13.7%)	48/714 (6.7%)		
50‑80	220/787 (27.9%)	41/73 (56.1%)	179/714 (56.1%)		
80‑100	342/787 (43.4%)	8/73 (10.9%)	334/714 (46.7%)		
> 100	18/787 (2.2%)	3/73 (4.1%)	15/714 (2.1%)		
**Admission HR**				0.005	55
< 50	94/762 (12.3%)	6/72 (8.3%)	88/690 (12.7%)		
50‑100	202 (26.5%)	15/72 (20.8%)	187/690 (27.1%)		
100‑120	154/762 (20.2%)	26/72 (36.1%)	128/690 (18.5%)		
> 120	312/762 (40.9%)	25/72 (34.7%)	287/690 (41.6%)		
Arrhythmia on admission, *n* (%)	208/755 (27.8%)	10/72 (13.9%)	198/683 (29%)	0.006	62
Pupilary response, *n* (%)	447/756 (59.1%)	63/73 (86.3%)	384/683 (56.2%)	< 0.001	61
Admission INR, median (IQR)	1.4 (1.2‑1.7)	1.4 (1.2‑1.6)	1.4 (1.2‑1.8)	0.9	355
Admission pH, median (IQR)	7.16 (7.01‑7.26)	7.19 (7.03‑7.28)	7.16 (7.01‑7.26)	0.5	229
Admission Lactate, median (IQR)	7.2 (4.3‑11.3)	5.9 (4.4‑11.02)	7.2 (4.3‑11.3)	0.5	255
PRBCs in first 24 h, median (IQR)	12 (6‑23)	6 (4‑10)	13 (6.5‑24)	< 0.001	64
FFP in first 24 h, median (IQR)	10 (4‑20)	5.5 (3‑9)	10 (4‑20)	0.001	83
Platelets in first 24 h, median (IQR)	3 (1‑10)	6 (0‑10)	3 (1‑10)	0.7	116
Laparotomy, *n* (%)	476/774 (61.5%)	48/72 (66.6%)	428/702 (61%)	0.3	43
Pelvic Surgery, *n* (%)	148/678 (21.8%)	9/73 (12.3%)	139/605 (22.9%)	0.03	139
Embolization, *n* (%)	105/707 (14.8%)	2/73 (2.7%)	103/634 (16.2%)	0.002	110

Table 2 presents REBOA procedure-related information and outcomes. REBOA practice patterns were significantly different between the groups. Patients from HICs got their REBOA inserted more often in the emergency room (*n* = 520/710, 73.2%); in contrast, a higher proportion of patients from LMICs underwent REBOA insertion in the operating room (*n* = 44/73, 60.2%) (*p* < 0.001). While femoral artery cannulation by a blind percutaneous approach was the preferred access technique in HICs (*n* = 370/721, 51.3%), surgical cutdown was more common in LIMCs (*n* = 43/73, 58.9%). Access guided by ultrasound was performed in 30.6% and 21.9% of patients from HICs and LMICs, respectively. Overall, the majority of the REBOAs were deployed by a trauma surgeon (547/727, 75.2%). However, the participation of radiologists and anesthesiologists in the insertion and deployment was more frequent in HICs.

**Table 2:** REBOA use and outcomes between LMICs and HICs

Variable	All (***n*** = 817)	LMIC (***n*** = 73)	HIC (***n*** = 744)	***p*** value	Missing
**REBOA procedure**					
**Access location**				< 0.001	34
ER, *n* (%)	548/783 (70%)	28/73 (38.3%)	520/710 (73.2%)		
OR, *n* (%)	214/783 (27.3%)	44/73 (60.2%)	170/710 (24.9%)		
Angio, *n* (%)	21/783 (2.6%)	1/73 (1.3%)	20/710 (2.8%)		
**Access technique**				< 0.001	23
Blind, *n* (%)	383/794 (48%)	13/73 (17.8%)	370/721 (51.3%)		
US guided, *n* (%)	237/794 (29.8%)	16/73 (21.9%)	221/721 (30.6%)		
Cut down, *n* (%)	162/794 (20.4%)	43/73 (58.9%)	119/721 (16.5%)		
Fluoroscopy, *n* (%)	12/794 (1.5%)	1/73 (1.37)	11/721 (1.53%)		
Cath/sheath diameter					454
**Primary performer**				< 0.001	90
Vascular surgeon, *n* (%)	53/727 (7.2%)	17/70 (24.2%)	36/657 (5.1%)		
Radiologist, *n* (%)	26/727 (3.5%)	1/70 (1.4%)	25/657 (3.8%)		
ER physician, *n* (%)	70/727 (9.6%)	6/70 (8.5%)	64/657 (9.7%)		
Trauma surgeon, *n* (%)	547/727 (75.2%)	43/70 (61.4%)	504/657 (76.7%)		
General surgeon, *n* (%)	7/727 (0.9%)	3/70 (4.2%)	4/657 (0.6%)		
Anesthesiologist, *n* (%)	13/727 (13%)	0/70 (0%)	13/657 (1.9%)		
Resident/fellow, *n* (%)	11/727 (1.5%)	0/70 (0%)	11/657 (1.6%)		
SBP at REBOA initiation, median (IQR)	60 (40‑80)	50 (32‑60)	62 (40‑80)	< 0.001	76
**Zone of occlusion**				0.02	10
Zone I, *n* (%)	555/807 (68%)	59/73 (80.8%)	496/734 (67.5%)		
Zone II, *n* (%)	16/807 (1.9%)	2/73 (2.7%)	14/734 (1.9%)		
Zone III, *n* (%)	236//807	12/73 (16.4%)	224//734 (30.5%)		
Confirmed balloon migration, *n* (%)	39/817 (4.7%)	1/73 (1.3%)	38/681 (5.1%)	0.2	0
Groin access complications, *n* (%)	65/775 (8.3%)	6/70 (8.5%)	59/705 (8.3%)	0.9	42
AKI, *n* (%)	137/743 (18.4%)	8/73 (10.9%)	129/670 (19.5%)	0.08	74
MODS, *n* (%)	130/729 (17.8%)	45/68 (66.1%)	85/661 (12.8%)	< 0.001	88
Respiratory failure, *n* (%)	95/727 (13.1%)	22/70 (31.2%)	73/657 (11.1%)	< 0.001	90
Sepsis, *n* (%)	82/729 (11.2%)	9/70 (12.8%)	73/659 (11.1%)	0.6	88
Ventilator days, median (IQR)	2 (1‑7)	3 (1‑6)	2 (1‑6)	0.4	89
Mortality, *n* (%)	479 (58.6%)	37 (50.6%)	442 (59.4%)	0.1	0

Patients from LMICs had a significantly higher occurrence of MODS (LMICs = 45/68, 66.1% vs. HICs = 85/661, 12.8%; *p* < 0,001), and respiratory failure (LMICs = 22/70, 31.2% vs. HICs = 73/657, 11.1%; *p* < 0,001). There were no differences in the occurrence of acute kidney injury, sepsis, and groin access complications. Similarly, there were no differences in ventilator days and mortality between groups.

Table 3 presents the general characteristics, hemodynamics parameters, surgical and resuscitation strategies, REBOA procedure-related information, and outcomes for the propensity-matched cohort.

**Table 3:** General characteristics, hemodynamics parameters, surgical and resuscitation strategies, REBOA procedure-related information, and outcomes for the propensity-matched cohort

Variable	All (***n*** = 132)	LMIC (***n*** = 66)	HIC (***n*** = 66)	***p*** value	Missing
Age, median (IQR)	35.5 (25‑51.5)	36 (27‑49)	34.5 (24‑57)	0.5	0
Gender [female], *n* (%)	32 (24.2%)	17 (25.7%)	15 (22.7%)	0.6	0
**Trauma mechanism**				0.1	0
Blunt, *n* (%)	82 (62.1%)	37 (56%)	45 (68.1%)		
Penetrating, *n* (%)	50 (37.9%)	29 (44%)	21 (31.9%)		
ISS, median (IQR)	32 (25‑48)	32 (25‑48)	32.5 (25‑45)	0.7	
Pre-hosp CPR, *n* (%)	4/129 (3.1%)	1/64 (1.5%)	3/65 (4.6)	0.6	3
CPR on arrival, *n* (%)	3 (2.3%)	1 (1.5%)	2 (3%)	1	0
**Admission SBP**				0.1	0
Not measurable	16 (12.1%)	9 (13.6%)	7 (10.6%)		
< 50	16 (12.1%)	9 (13.6%)	7 (10.6%)		
50‑80	80 (60.6%)	39 (59.1%)	41 (62.1%)		
80‑100	9 (6.8%)	7 (10.6%)	2 (3%)		
> 100	11 (8.3%)	2 (3%)	9 (13.6%)		
**Admission HR**				0.01	3
< 50	5/129 (3.8%)	4/65 (6.15%)	1/64 (1.5%)		
50‑100	24/129 (18.6%)	12/65 (18.4%)	12/64 (18.7%)		
100‑120	37/129 (28.6%)	25/65 (38.4%)	12/64 (18.7%)		
> 120	63/129 (48.8%)	24/65 (36.9%)	39/64 (60.9%)		
**Admission HR ≥ 100,** ***n*** **(%)**	100/129 (77.5%)	49/65 (75.3%)	51/64 (79.6%)	0.5	3
Arrhythmia on admission, *n* (%)	18/115 (15.6%)	7/66 (10.6%)	22/49 (22.4%)	0.08	17
Pupilary response, *n* (%)	115 (87.1%)	59 (89.3%)	56 (84.8%)	0.4	0
Admission INR, median (IQR)	1.36 (1.2‑1.6)	1.37 (1.23‑1.62)	1.35 (1.2‑1.6)	0.3	53
Admission pH, median (IQR)	7.19 (7.03‑7.28)	7.21 (7.03‑7.28)	7.19 (7‑7.27)	0.9	22
Admission lactate, median (IQR)	6.8 (4‑10.8)	5.8 (4.3‑10.8)	7.2 (4‑10.4)	0.4	33
PRBCs in first 24 h, median (IQR)	7 (4‑10)	6 (4‑10)	8 (4‑12)	0.3	0
FFP in first 24 h, median (IQR)	5 (2‑10)	6 (3‑9)	5 (2‑10)	0.4	4
Platelets in first 24 h, median (IQR)	2 (0‑6)	6 (0‑10)	2 (0‑4)	0.1	17
Laparotomy, *n* (%)	88/130 (67.7%)	45/65 (69.2%)	43/65 (66.1%)	0.7	2
Pelvic surgery, *n* (%)	22/121 (18.2%)	9/66 (13.6%)	13/55 (23.6%)	0.1	11
Embolization, *n* (%)	14/122 (11.4%)	2/66 (3%)	12/56 (21.4%)	0.001	10
**REBOA procedure**					
**Access location**				0.09	
ER, *n* (%)	59 (44.7%)	24 (36.3%)	35 (53%)		
OR, *n* (%)	71 (53.8%)	41 (62.1%)	30 (45.4%)		
Angio, *n* (%)	2 (1.5%)	1 (1.5%)	1 (1.5%)		
**Access technique**				< 0.001	1
Blind, *n* (%)	48/131 (36.6%)	12/66 (18.1%)	36/65 (55.3%)		
US guided, *n* (%)	38/131 (29%)	15/66 (22.7%)	23/65 (35.4%)		
Cut down, *n* (%)	43/131 (32.8%)	38/66 (57.5%)	5/65 (7.6%)		
Fluoroscopy, *n* (%)	2/131 (1.5%)	1/66 (1.5%)	1/65 (1.5%)		
**Primary performer**				0.001	11
Vascular surgeon, *n* (%)	18/121 (14.9%)	14/64 (21.8%)	4/57 (7%)		
Radiologist, *n* (%)	7/121 (5.8%)	1/64 (1.5%)	6/57 (10.5%)		
ER physician, *n* (%)	7/121 (5.8%)	6/64 (9.3%)	1/57 (1.7%)		
Trauma surgeon, *n* (%)	82/121 (67.7%)	40/64 (62.5%)	42/57 (73.6%)		
General surgeon, *n* (%)	3/121 (2.4%)	3/64 (4.6%)	0/57 (0%)		
Anesthesiologist, *n* (%)	3/121 (2.5%)	0/64 (0%)	3/57 (5.2%)		
Resident/fellow, *n* (%)	1 (2.5%)	0/64 (0%)	1/57 (1.7%)		
SBP at REBOA initiation, median (IQR)	60 (40‑70)	50 (32‑65)	63 (50‑80)	< 0.001	6
**Zone of occlusion**				0.1	0
Zone I, *n* (%)	98 (74.2%)	53 (80.3%)	45 (68.1%)		
Zone II, *n* (%)	3 (2.2%)	2 (3%)	1 (1.5%)		
Zone III, *n* (%)	31 (23.4%)	11 (16.7%)	20 (30.3%)		
Confirmed balloon migration, *n* (%)	2 (1.5%)	1 (1.5%)	1 (1.5%)	1	0
Groin access complications, *n* (%)	16/125 (12.8%)	6/64 (9.3%)	10/61 (16.3%)	0.2	7
AKI, *n* (%)	21/129 (16.2%)	8/66 (12.1%)	13/63 (20.6%)	0.1	3
MODS, *n* (%)	54/124 (43.4%)	43/62 (69.3%)	11/62 (17.7%)	< 0.001	8
Respiratory failure, *n* (%)	33/125 (26.4%)	22/64 (34.4%)	11/61 (18%)	0.03	7
Sepsis, *n* (%)	15/125 (12%)	9/64 (14%)	6/61 (9.8%)	0.4	7
Ventilator days, median (IQR)	3 (1‑9)	3,5 (1‑9)	3 (1‑9)	0.6	5
Mortality, *n* (%)	57 (43.1%)	31 (49.9%)	26 (39.4%)	0.3	0

After the 1:1 propensity matching, LMICs (*n* = 66) and HICs (*n* = 66) groups were adequately balanced in the majority of their baseline characteristics. However, there were still significant differences in the rate of IR angioembolization (LMICs = 2/66, 3% vs. HICs = 12/56, 21.4%; *p* = 0.001), the backgrounds of providers deploying REBOA, and the preferred access technique. Blind percutaneous approach and surgical cutdown were the preferred means of femoral cannulation in HICs (55.3%) and LIMCs (57.5%), respectively. Also, patients from LMICs had significantly lower values of SBP at REBOA initiation (SBP at REBOA initiation, median [IQR]: LMIC = 50 mmHg [32‑65] vs. HIC = 63 mmHg [50‑80]; *p* < 0.001).

The differences in the occurrence of MODS and respiratory failure persisted after the 1:1 propensity matching. A significantly higher proportion of patients from LIMCs presented these outcomes compared to patients from HICs. There were no differences in the occurrence of sepsis, acute kidney injury, and mortality between the propensity score-matched groups.

After adjusting by tachycardia (HR ≥ 100), procedure (laparotomy or pelvic surgery), the value of systolic blood pressure at REBOA initiation, and the need for massive transfusion (≥ 10 PRBCs in the first 24 h), logistic regression showed no differences in mortality for LMICs when compared to HICs; neither in the non-matched cohort (OR = 0.63; 95% CI 0.36‑1.09; *p* = 0.1) nor in the matched cohort (OR = 1.45; 95% CI 0.63‑3,33; *p* = 0.3).

## Discussion

This study, which reflects the worldwide practice patterns of REBOA for trauma patients by country income group, revealed considerable variation in the management practices of this intervention and the peri-interventional surgical maneuvers used for its insertion and deployment between high and low-to-middle income countries. In addition to the variations in REBOA clinical practice patterns, we found significant differences in the clinical presentations, resuscitation strategies, surgical interventions, and some clinically relevant outcomes; however, with no differences in the risk-adjusted odds of mortality between the groups analyzed.

To our knowledge, this is the first study providing global insight into the use of REBOA as an adjunct for hemorrhage control and resuscitation in relation to the income of countries, and these results should encourage physicians using REBOA to step up efforts toward standardizing the practice of this endovascular strategy in order to treat patients similarly. Therefore, resulting in similar treatment patterns and surgical outcomes.

We found no differences in the risk-adjusted odds of mortality; however, patients from LMICs did have a significantly higher frequency of MODS and respiratory failure that persisted even after propensity score matching. Although previous studies have shown that REBOA can have a significant effect on the outcomes mentioned above [1, 14], the differences in pre-hospital medical services between LMICs and HICs offer a more plausible explanation for the results observed. The fact that patients from LMICs had worse outcomes in terms of organ failure may be due, in part, to the pervasive deficiencies in LMICs pre-hospital trauma care [15, 16], which is often limited by inadequate flow of transportation and lack of protocols for field triage and standards of pre-hospital care. Indeed, patients from LMICs included in this study were less likely to receive pre-hospital and admission CPR. The latter situations could be translated into an inadequate pre-hospital resuscitation of trauma patients, which may result in a poor physiological status on admission and, thereby, a higher chance of organ failure during the hospital stay.

On the other hand, although our finding on mortality is not surprising, it should be interpreted cautiously. The same deficiencies in LMICs pre-hospital care could have introduced significant survivorship bias, meaning that in LMICs, the patients with a higher chance of survival arrived in the emergency department and had the opportunity to get a REBOA. Improving pre-hospital care in LIMCs while simultaneously implementing endovascular trauma management should be a priority for trauma stakeholders to further the field of trauma care globally.

What is surprising is that the hospital scenario for REBOA insertion and techniques for femoral artery cannulation varied widely between the groups analyzed. While in LMICs, the insertion and deployment of REBOA occurred more frequently in the operating room and by surgical cutdown, in HICs, these procedures were performed more often in the emergency room and by blind percutaneous insertion. Previous studies have shown a higher incidence of groin access complications when REBOA is inserted by surgical cutdown [17], and some authors recommend in favor of performing groin access in an operating room controlled setting [18]. Therefore, our findings are of concern because a lack of standardization of surgical procedures for REBOA insertion and deployment can jeopardize clinical outcomes and patient safety. We urge a shift in REBOA surgical practice toward ultrasound-guided access, with surgeons adopting a more cautious, safe, and systematic approach to REBOA insertion and deployment. To this end, trauma surgeons using this endovascular technology should follow published guidelines and recommendations [19, 20]; however, they should be prepared to adapt these guidelines to make them appropriate for their local trauma-system reality and develop simple, scalable solutions to improve the delivery of REBOA, regardless of the income of their country of origin.

This study revealed that different surgeons are using different approaches for similar indications and modified surgical procedures when using the same endovascular intervention. For example, we found a significant difference in the use of IR angioembolization as a method for bleeding control, with higher use of this modality in HICs. Also, ultrasound was more frequently used during REBOA insertion in high-income countries, and the proportion of radiologists involved in REBOA use was higher in these countries. It can be inferred that the higher the income, the greater the capacity of the healthcare system to allocate resources to offer more advanced care with better technology. Therefore, it is rational to posit that these clinical practice behaviors are plausibly related to the income of the regions where trauma surgeons are practicing.

It is now clear that there is variability in the REBOA clinical practice patterns in trauma care between LMICs and HICs. Nevertheless, these differences can provide meaningful opportunities to examine and include the perspectives from trauma surgeons practicing in LMICs into the teams producing the guidelines for REBOA use. To date, some guidelines have been published in well-recognized international journals [19–21]. Of note, however, these guidelines often lack the representation of physicians from LMICs in their list of authors, and only a few studies performed in LMICs are included in the references supporting the guidelines. These disparities raise concerns that the challenges and problems on trauma care and REBOA implementation inherent to LMICs could be inadequately addressed [4, 10]. Moreover, the lack of inclusion of stakeholders from LMICs into REBOA clinical guidelines may be of greater concern because most of the world’s population lives in LMICs, where the burden of traumatic injuries is enormous, and outcomes are often poor [22]. Therefore, future international REBOA guidelines should aim to be more globally applicable, with dedicated recommendations for its use in LMICs. In this way, the practice and delivery of REBOA could be optimized globally and enhanced most favorably for all.

To bridge the gap between HICs and LMICs, trauma surgeons practicing either in low-volume centers or in low resource settings should implement policies to improve endovascular trauma management quality. These quality improvement policies should include a complete REBOA curriculum, including educational goals and assessment of learning outcomes.

### Limitations

Our study has its limitations, and results should be interpreted in the context of the datasets used and the analyses performed. First, our study was at risk of residual confounding because information on some clinically relevant variables was not collected. This lack of data granularity could affect risk adjustment, even after using propensity score matching. Second, the lack of uniformity of diagnostic and therapeutic approaches between the reporting centers may have affected the outcomes observed, further comprising the validity of our results and the interpretations derived from them. Third, because we did not perform sample size calculations, this study may not have adequate power to detect the true effect of the countries’ income on REBOA-treated patients.

Despite our limitations, we believe that the countries’ income can be used as a proxy for the health care services’ capacity of each country. Therefore, the REBOA outcomes information related to this exposure should provide essential insights on how trauma-system and hospital capacity planning could be improved and adapted to the implementation of novel endovascular approaches for bleeding control.

## Conclusion

There is considerable variation in the management practices of REBOA and the outcomes associated with this intervention between high and low-to-middle income countries. Trauma surgeons practicing REBOA around the world should joint efforts to standardize the practice of this endovascular technology worldwide.

## Data Availability

The datasets generated and/or analyzed during the current study are not publicly available but could be available from the corresponding author on reasonable request.

## Abbreviations

REBOA: Resuscitative endovascular balloon occlusion of the aorta
